# Chemokines in tumor progression and metastasis

**DOI:** 10.18632/oncotarget.1426

**Published:** 2013-11-09

**Authors:** Purvaba J. Sarvaiya, Donna Guo, Ilya Ulasov, Patrik Gabikian, Maciej S. Lesniak

**Affiliations:** ^1^ The Brain Tumor Center, The University of Chicago, Chicago, Illinois, USA

**Keywords:** Chemokines, chemokine receptors, cancer, metastasis

## Abstract

Chemokines play a vital role in tumor progression and metastasis. Chemokines are involved in the growth of many cancers including breast cancer, ovarian cancer, pancreatic cancer, melanoma, lung cancer, gastric cancer, acute lymphoblastic leukemia, colon cancer, non-small lung cancer and non-hodgkin's lymphoma among many others. The expression of chemokines and their receptors is altered in many malignancies and leads to aberrant chemokine receptor signaling. This review focuses on the role of chemokines in key processes that facilitate tumor progression including proliferation, senescence, angiogenesis, epithelial mesenchymal transition, immune evasion and metastasis.

## INTRODUCTION

Chemokines are a group of small molecular weight proteins that bind to the G protein coupled chemokine receptors [1]. Chemokines play an important role in cell migration, development, immune surveillance, inflammation as well as in many pathological conditions [1]. Chemokines play a key role in regulating immune response and inflammation by their involvement in the regulation of leukocyte trafficking and positioning. The binding of the chemokines to the receptors leads to a conformational change, which activates signaling pathways and promotes migration. Chemokines and their receptors are divided into four families based on the pattern of cysteine residues: CXC, CC, CX3C and C, where C represents the cysteine and X represents non-cysteine amino acids [2, 3]. Approximately 20 chemokine receptors and 50 chemokines have been identified in humans, which are listed in Table I. Chemokines can also be divided into two groups based on their function: inflammatory chemokines and homeostatic chemokines. As the names suggest, inflammatory chemokines are induced by inflammation while homeostatic chemokines are constitutively expressed and are involved in homeostatic immune regulation.

**Table I:** List of the chemokine receptors and chemokine ligands that bind to the receptors

Chemokine Receptor	Chemokine Ligands
CXCR1	CXCL6, CXCL8
CXCR2	CXCL1, CXCL2, CXCL3, CXCL5, CXCL7, CXCL8, CXCL6
CXCR3	CXCL4, CXCL9, CXCL10, CXCL11,
CXCR4	CXCL12
CXCR5	CXCL13
CXCR6	CXCL16
CXCR7	CXCL12, CXCL11
CCR1	CCL3, CCL4, CCL5, CCL7, CCL14, CCL15, CCL16, CCL23
CCR2	CCL2, CCL7, CCL8, CCL12, CCL13
CCR3	CCL5, CCL7, CCL11, CCL13, CCL15, CCL24, CCL26, CCL28
CCR4	CCL2, CCL3, CCL5, CCL17, CCL22
CCR5	CCL3, CCL4, CCL5, CCL8
CCR6	CCL20
CCR7	CCL19, CCL21
CCR8	CCL1, CCL4, CCL17
CCR9	CCL25
CCR10	CCL27, CCL28
CCR11	CCL2, CCL7, CCL8, CCL12, CCL13, CCL19, CCL21, CCL25
CX3CR1	CX3CL1
XCR1	XCL1, XCL2

Chemokine receptors are seven transmembrane spanning proteins coupled to G-protein-coupled-receptors (GPCRs). These receptors are named based on the chemokine ligands to which they bind [2, 3]. For example, CXC receptors (CXCR1, 2, 3, 4 and 5) bind CXC chemokines, CC receptors (CCR1, 2, 3, 4, 5, 6, 7, 8, 9) bind CC chemokines; CX3C receptor binds CX3C chemokine and lastly, the XC receptor binds the C chemokine. In spite of the fact that chemokine receptors bind to their specific chemokine sub-groups, there is significant ligand promiscuity. Some chemokines can bind to and signal three chemokine receptors. Also, responses to some chemokine receptors could be elicited by as many as 10 ligands [4]. Furthermore, it is important to consider that there are differences in the mouse and human chemokine families. For example, CXCL8, CCL18, CCL23 are present in humans but absent in mice [2, 5]. Hence, all of the observations in the mouse models cannot be generalized in humans. Furthermore, many post-translational modifications affect the chemokine receptor signaling, receptor specificity as well as chemotactic property of chemokines and thus affect their biological functions. Some of the post-translational modifications in chemokines include glycosylation, citrullination, and proteolytic processing at the N and C terminus [6-9].

Chemokines play an important role in the progression of cancers. They are involved in tumor growth, senescence, angiogenesis, epithelial mesenchymal transition, metastasis and immune evasion. The expression of chemokines and their receptors is altered in many malignancies and subsequently leads to aberrant chemokine receptor signaling. This alteration occurs due to inactivation of the tumor suppressor genes or constitutive activation of the oncogenes that play a role in the regulation of the chemokines. Furthermore, deregulated expression of the transcription factors also affects the levels of chemokine and receptors regulated by them and promotes tumorigenesis. For example, the nuclear factor-kappa B (NF-kappa B) family of transcription factors regulates the expression of many chemokines [10]. NF-kappa B is constitutively activated in many tumors which leads to the constitutive expression of the chemokines regulated by them which then promotes tumorigenesis [10]. In this review, we will discuss the role of chemokines in tumor growth, progression, and metastasis.

### Chemokines in Senescence

Cellular senescence is a state of growth arrest that prevents unlimited proliferation of the cells [11]. Thus, cellular senescence is an important mechanism and it has attracted the attention of cancer scientists because of its ability to protect normal cells from transforming into cancer cells. Interestingly, oncogenes play an important role in inducing senescence [12]. Oncogene induced senescence (OIS) prevents unlimited cell proliferation and contributes toward preventing oncogenic transformation of the cells. Tumorigenesis is correlated with the secretion of cytokines and chemokines. Of note, some studies described below demonstrate that chemokines and their receptors promote senescence and delay tumorigenesis. IL-1α plays an important role in the production of senescence associated chemokine CXCL8 along with IL-6 [13]. However, the loss of chemokine receptor CXCR2 reduces oncogene induced senescence along with the DNA damage response [14]. The activation of the transcription factors NF-kappaβ and C/EBPβ contributes to the secretion of CXCR2 binding chemokines and IL-6 [14, 15]. Also, the reintroduction of the chemokine receptor CXCR2 leads to premature senescence by a p53 dependent mechanism [14]. This suggests that the CXCR2 along with the chemokines that bind to it promote cellular senescence and delay the process of oncogenesis. Low levels of CXCR2 are also found in head and squamous cell carcinoma [16]. However, mutations in CXCR2 or downregulation of CXCR2 expression may affect the ability of this chemokine receptor to induce senescence. One inactivating mutation of CXCR2 was found in NCI-H1395 cell line [17]. The inability to induce senescence by mutated CXCR2 may in fact promote tumorigenesis instead of blocking it. The above studies demonstrate the importance of CXCR2 in cellular senescence. Future studies should aim to determine the role of other chemokine receptors in cellular senescence.

### Chemokines in epithelial mesenchymal transformation

An epithelial mesenchymal transition (EMT) is a biological process in which an epithelial cell undergoes biochemical changes to assume a mesenchymal cell phenotype that has increased capacity for migration and invasion [18]. Cancer cells acquire the mesenchymal phenotype to migrate, invade and metastasize. The chemokine CXCL8 is induced in cells that undergo TGF-β driven EMT. The chemokine CXCL8 binds to the chemokine receptor CXCR1. Interestingly, CXCR1 is upregulated during EMT, thus demonstrating that the regulated expression of the chemokine CXCL8 along with its receptor CXCR1 is linked to EMT [19]. The treatment of rat hepatoma cells with TGF-β promotes EMT phenotype in the cells. TGF-β upregulates the expression of CXCR4 and the cells with a mesenchymal phenotype exhibit higher levels of CXCR4. Furthermore, the knockdown of CXCR4 in these cells or treatment with CXCR4 antagonist leads to a decrease in cell migration. This study suggests that CXCR4 induction after treatment with TGF-β may play an important role in promoting EMT and thus aid in migration of these cells [20]. Another study also demonstrates the importance of CXCL8/CXCR1 in inducing EMT. The induction of EMT in human carcinoma cells overexpressing the transcription factor Brachyury is associated with increased secretion of multiple chemokines including CXCL8, CCL5 and CXCL1. However, the induction of CXCL8/CXCR1 axis is critical for EMT. The authors suggest that inhibiting CXCL8 signaling may help inhibit EMT by targeting the cells with the mesenchymal and invasive phenotype [21]. A recent study demonstrated that using a CXCR7 antagonist, CCX733 drastically reduced EMT in bladder cancer, which suggests the importance of CXCR7 in regulating EMT and in the development of bladder cancer [22]. Furthermore, in human colorectal carcinoma cells, the transcription factor SNAIL which plays a critical role in regulating EMT also regulates the expression of the chemokine CXCL8, which further demonstrates the importance of CXCL8 or IL-8 in the process of EMT in cancer [23]. These studies demonstrate the importance of chemokine receptor signaling in EMT which is an essential precursor for metastatic transformation of cancer cells. However, more studies are required to determine if targeting chemokine receptors can prevent EMT in various cancer types.

### Chemokines in tumor growth

A number of studies have demonstrated the role of chemokines and chemokine receptors in tumor growth. Chemokines activate MAPK/ERK signaling pathway and thus promote tumor cell proliferation. The chemokine CXCL12 is involved in the growth of many cancers including acute lymphoblastic leukemia, chronic B cell leukemia, glioma, breast cancer, ovarian cancer, small cell lung cancer, non-Hodgkin's lymphoma and colon cancer [24-32]. Also, the chemokines CXCL1, CXCL2 and CXCL3 have been shown to play a role in the growth of pancreatic cancer, melanoma, lung cancer, adenocarcinoma and gastric cancer [33, 34]. The chemokine receptor CXCR4 is over-expressed in many cancer types and is also involved in their proliferation. These cancers include melanoma, breast cancer, ovarian cancer, prostate cancer, lung cancer, glioma, renal cancer, B-cell chronic lymphocytic leukemia and thyroid cancer [28, 35-43]. The inhibition of the chemokine receptor CXCR4 leads to apoptosis in ovarian cancer, hepatoma and chronic lymphocytic leukemia cells, thus demonstrating the importance of this receptor in cell survival and proliferation [20, 44, 45]. Another chemokine receptor CXCR2 plays a vital role in the proliferation of esophageal cancer cells via GROα and GROβ [46]. Furthermore, the chemokine receptors for CXCL8: CXCR1 and CXCR2 are expressed in human gastric carcinoma cells and play a role in the proliferation of the cancer cells [47]. Overexpression of the receptors CXCR1 and CXCR2 in human melanoma cells increased cell proliferation and invasion *in vitro* and significantly enhanced tumor growth *in vivo*, thus demonstrating the importance of these receptors in melanoma tumor growth and progression [48]. The chemokine receptor CCR6 is expressed in colorectal cancer and stimulation by the chemokine ligand CCL20 promotes proliferation of colorectal cancer cells [49]. Furthermore, the expression of CXCR6 and its ligand CXCL16 in prostate cancer correlated with prognosis and CXCL16 enhanced the growth of CXCR6 expressing cancers [50, 51]. CXCR6 along with CXCL16 mediates pro-tumorigenic effect on prostate cancer cells by inducing the migration and proliferation of tumor associated leukocytes [50]. The chemokine receptor CCR10 is expressed on melanoma cells. The exposure of the ligand CCL27 to the melanoma cells leads to activation of Akt and phosphatidylinositol-3-kinase and protects the cells from apoptosis. Thus, CCL27/CCR10 contributes to the growth of melanoma cells by the activation of PI3K/Akt pathway and by evading the host anti-tumor response [52]. The cells of squamous cell carcinoma of head and neck secrete the chemokines CCL19 and CCL21, which leads to the activation of CCR7 and promotes tumor growth and progression [53].

On the other hand, some chemokine receptors inhibit the proliferation of tumor cells. For example, the chemokine receptor CCR1 can reduce the proliferation of human hepatocellular carcinoma cells [54]. Also, the inhibition of CCR5 increased the proliferation of breast cancer cells bearing wild type p53, which suggests that CCR5 inhibits breast cancer progression in a p53-dependent manner [55]. Hence, given the pro-and anti-tumorigenic role of chemokines and its receptors, more studies are required to determine why some chemokine receptors facilitate tumor progression and others inhibit the growth of the tumors. Also, future studies should focus on elucidating the mechanism by which chemokines and chemokine receptors promote tumorigenesis, which could lead to targeted therapies.

### Chemokines in angiogenesis

Angiogenesis, the process of formation of new blood vessels from existing blood vessels is critical in tumor progression. Many chemokines and chemokine receptors play a vital role in angiogenesis. Chemokines can either stimulate the expression of pro-angiogenic factors or can bind to the chemokine receptors on the surface of endothelial cells and promote angiogenesis. The ELR motif present in the CXC chemokines plays an important role in promoting angiogenesis [56]. The CXC chemokines that have the ELR motif are potent angiogenic factors whereas chemokines without the ELR motif promote angiostasis [56]. The chemokines CXCL1, CXCL2, CXCL3, CXCL5, CXCL6, CXCL8, CXCL12, CCL2, CCL11 and CCL16 are angiogenic chemokines whereas CXCL4, CXCL9, CXCL10, CXCL11 and CXCL14 are angiostatic chemokines [57, 58].

The reduction in the levels of chemokines CXCL1, CXCL2 and CXCL3 in melanoma results in a decrease in tumor angiogenesis and tumor growth, suggesting the importance of these chemokines in angiogenesis [59, 60]. The chemokine receptors CXCR1, CXCR2 and CXCR4 are expressed on the endothelial cells [61, 62]. The knockdown of CXCR1 and CXCR2 in human microvascular endothelial cells reduced migration, invasion and capillary like structure formation demonstrating the importance of these receptors in angiogenesis [61]. Also, the chemokine CXCL12 activates the chemokine receptor CXCR4 on endothelial cells, which promotes endothelial cell migration and proliferation in ovarian cancer cells [63]. The chemokine receptor CCR2 is also expressed by the endothelial cells [64]. A recent study demonstrates the role of CCR2-CCL2 axis in angiogenesis as well as tumor survival [65].

### Chemokines that inhibit angiogenesis

The chemokine receptor CXCR3 on human microvascular endothelial cells (HMVEC) blocks the proliferation of HMVEC *in vitro* and this effect is inhibited by anti-CXCR3 antibody. This study demonstrates the angiostatic effect of the chemokine receptor CXCR3 [66]. As mentioned previously, CXCL9, CXCL10 and CXCL11 (ELR^−^ chemokines) are the anti-angiogenic chemokines and they inhibit angiogenesis via the chemokine receptor CXCR3-B [67, 68]. These chemokines inhibit angiogenesis in colon carcinoma, melanoma and uterine cervical cancers [69-71]. Also, the chemokine CXCL4 has been shown to have angiostatic effects [72]. The enhanced expression of the duffy antigen on endothelial cells suppresses the angiogenic effect of the CXC chemokines [73]. The fine balance between the angiogenic as well as angiostatic chemokines determines the fate of tumor cells by regulating angiogenesis.

Moreover, chemokines could also regulate angiogenesis indirectly. For example, CXCL6 and CXCL8, which are ELR^+^ chemokines, promote angiogenesis by recruiting neutrophils [74]. The production of CXCL6 by the endothelial cells contributes to angiogenesis by attracting neutrophils that promote matrix degradation [75]. In a mouse model of pancreatic cancer, neutrophils contribute to angiogenesis by recruiting MMP-9 that activates vascular endothelial growth factor (VEGF) which is critical for angiogenesis [76]. Chemokines also promote angiogenesis by recruiting leukocytes, myeloid derived suppressor cells (MDSC), dendritic cells and tumor associated macrophages (TAM) [61, 77-79]. The chemokine CCL2 aids in the recruitment of these cells. The recruited TAMs and MDSCs could acquire characteristics of the endothelial cells and thus promote angiogenesis by being a part of the vasculature [80]. The recruited cells can also produce angiogenic factors such as vascular endothelial growth factor (VEGF), platelet derived growth factor (PDGF), transforming growth factor beta (TGFβ), chemokines such as CXCL8, as well as matrix metalloproteinases such as MMP-2 and MMP-9 [10, 78, 81].

### Chemokines in metastasis

Metastasis is the process during which malignant tumor cells leave the primary tumor site, enter the blood stream or lymphatic system and migrate to other organs or sites in the body. Metastasis is a non-random, sequential, and organ-specific process. Certain organs, such as the liver, lungs, brain, lymph nodes, and bone marrow are common sites of metastasis, while others, such as the kidney, pancreas, and skin are rare [82]. Metastasis is the leading cause of death for a majority of solid tumors, and the ability to metastasize is one of the key features which distinguishes malignant tumors from benign lesions. While metastasis is a complex process involving various factors and small molecule regulators, studies have suggested that chemokines and their receptors play a key role in metastasis [38].

In normal physiological functions, homeostatic chemokines regulate the migration of leukocytes by recruiting specific populations of lymphoid cells to certain tissues in either innate or acquired immune responses. For instance, chemokine ligand CCL27 induces migration of leukocyte antigen CLA+ T cells which express chemokine receptor CCR10 to the skin [83], and ligand CXCL12 in the bone marrow recruits hematopoietic stem cells which express the receptor CXCR4 [38]. While chemokines normally regulate the migration of immune cells, other cell types can take advantage of these chemokine “pathways” by expressing the appropriate receptor. Recent studies suggest that metastatic cancer cells simply co-opt these chemokine pathways to migrate to distant sites. Like normal leukocyte migration, tumor cell metastasis requires passage through vascular barriers, entry into the circulation, and extravasation at distant, non-random, organ-specific locations. Since leukocyte trafficking is regulated by chemokine receptors and their ligands, chemokines may also play a key role in initiating and regulating tumor cell migration and metastasis.

#### a CXCR4-CXCL12

The CXCR4/CXCL12 axis is one of the most studied chemokine receptor axis and has been shown to play a vital role in metastasis. Studies show that metastatic breast cancers selectively express CXCR4 and migrate to organs that express high levels of its respective ligand CXCL12, also known as SDF-1 [38]. Chemokine receptor CXCR4 is consistently upregulated in metastatic breast cancer cell lines, lymph node metastases, and liver metastases while expression levels are undetectable in normal epithelial cells [38]. Its ligand CXCL12, meanwhile, is preferentially expressed in the most common sites of breast cancer metastasis, lung, brain, lymph nodes, liver, and bone marrow [38, 84]. This suggests that metastatic breast tumor cells selectively express CXCR4 which leads them to organs with high expression levels of CXCL12. Moreover, *in vivo* inhibition of CXCR4-CXCL12 interactions significantly reduces metastasis of breast tumor cells to the lymph node and lungs [38]. In addition, CXCR4-CXCL12 interactions also induce migratory responses that give tumor cells invasive ability. CXCR4-CXCL12 receptor-ligand interactions in breast cancer trigger actin polymerization which allows tumor cells to invade neighboring tissues and successfully metastasize [85], form pseudopodia, induce directional invasion of breast tumor cells [38]. Inhibition of CXCL12-CXCR4 interactions using anti-CXCR4 or CXCL12 antibodies significantly impairs these migratory responses by 63-76% and 60-62%, respectively [38].

Additional studies demonstrate that *de novo* expression of CXCR4 is sufficient to increase tumor invasion and metastasis in an organ-specific manner [86]. The murine B16 melanoma cell line experiences marked increase in metastasis to the lung when transfected with CXCR4 [86]. Inhibition of CXCR4 by the small molecule antagonist T22, however, stops the increase in metastasis of CXCR4-B16 cells [86]. Murkami *et. al* not only confirm the importance of CXCR4-CXCL12 in mediating metastasis, but they demonstrate that CXCR4 expression is sufficient for metastasis to occur [86].

Many metastatic human prostate cancers also express functional CXCR4 [87]. CXCR4 expression in prostate cancer enhances the invasive, metastatic ability of tumor cells in the presence of CXCL12 ligand, while inhibition of CXCR4 decreases metastatic ability [36, 87]. In comparison, presence of CXCL12 did not affect migratory ability of normal prostate epithelial cells [87]. Additionally, CXCR4 mediates prostate tumor cell adhesion through the α5 and β3 integrins [88]. High expression levels of CXCR4 in human prostate cancers are associated with more frequent local recurrence and distant metastasis, suggesting that CXCR4 not only serves as a potential therapeutic target, but also a prognostic marker [89, 90].

Interestingly, high levels of CXCR4 are also observed in CD44^+^/CD133^+^ prostate cancer stem cells (CSC) [91]. Since CSCs can initiate metastasis and help form new or recurrent tumors after initial treatment [92], the CXCR4-CXCL12 pathway may indirectly promote metastasis by maintaining and promoting stemness of CSCs [91]. Studies show that increased CXCR4 and CXCL12 expression promotes adhesion of CD133+/CD44+ prostate CSCs to the extracellular protein fibronectin, important for distal organ seeding and initiation of secondary tumors [91]. In addition, CXCL12 causes activation of the PI3K pathway and promotes proliferation of CXCR4^+^ prostate CSCs [91]. Thus, the active CXCR4-CXCL12 pathway in prostate CSCs may indirectly promote metastasis by regulating cell adhesion, proliferation, differentiation potential, and tumorigenicity of prostate CSCs [91].

Studies show that CXCR4 plays an important role in metastasis of many cancers including breast [93-95], lung [96, 97], colorectal [98-100], gastric [101-103], ovarian [104, 105], prostate [106], pancreatic [107, 108], melanoma [100, 109-111], esophageal [112, 113], head and neck [114], bladder [115], osteosarcoma [116], neuroblastoma [117], glioblastoma [118, 119], and acute lymphoblastic leukemia [120]. In breast cancer, CXCR4 promotes metastasis to the lungs, liver, and lymph nodes [38]. In gastric cancer, studies show that CXCR4 promotes metastasis to the lymph nodes [102, 103]. Also, a study demonstrates that CXCR4 expression in esophageal cancer enhances metastasis to the lymph nodes and bone marrow [112]. Additional studies show that the CXCR4/CXCL12 axis in ovarian cancer may promote peritoneal metastasis [104] as well as metastasis to the lymph nodes [121]. CXCR4 may also play a role in the metastasis of osteosarcoma to the lung [116] and neuroblastoma metastasis to the bone and bone marrow [117]. Thus, a number of studies have confirmed the important role of CXCR4 in metastasis.

#### b CCR7-CCL19/CCL21

CCR7-mediated leukocyte migration is extremely important in normal immune responses. CCR7 recruits naive T cells and activated dendritic cells to the lymph nodes, where they engage with one another and initiate adaptive immune responses [122]. CCR7 has two chemokine ligands: CCL19 and CCL21 [123, 124]; CCL21 regulates naive T-cell homing to secondary lymphoid organs, while CCL19 activates T-cells [125]. Loss of CCL21 or inhibition of CCR7 gene results in significantly impaired T-cell homing to secondary lymphoid organs [126]. Many studies suggest that tumor cells co-opt the normal mechanism of CCR7-CCL21 leukocyte homing to metastasize to the lymph nodes. Studies show a majority of primary breast cancer tissues and metastatic cancer cells in the lymph nodes express CCR7, and there is significant correlation between CCR7 expression and lymph node metastasis. In addition, higher CCR7 expression is correlated with lower survival and worse prognosis in breast cancer patients [127]. Correlation between lymph node metastasis and CCR7 expression is observed in many cancers including esophageal squamous cell carcinoma [128], melanoma [129], non-small cell lung [130], head and neck [131], gastric [132], and colorectal [133].

*De novo* expression of CCR7 is sufficient to induce preferential metastasis to the lymph nodes in several human and mouse breast tumor cell lines that, without CCR7 expression, normally metastasize solely to the lung [134]. Expression of CCR7 in a non-metastatic B16 melanoma cell line also increases metastasis to the lymph nodes [135]. Interestingly, expression of CCR7 in B16 melanoma cells induces metastasis to the lymph nodes [135], while, as previously discussed, expression of CXCR4 in murine B16 cells increases metastasis to the lungs [86]. CCR7 expression in metastatic melanoma also induces *in vivo* growth toward lymphatic endothelial cells [136]. High CCR7 expression in human non-Hodgkin's lymphoma induces metastatic spread through the PI3K/Akt signal pathway [137]. CCR7 expression also induces lymphatic spread in human pancreatic ductal adenocarcinoma [138]. In colon cancer, a study suggests that CCR7 promotes metastasis by upregulating matrix metalloproteinase-9 (MMP-9) expression [139].

Additional studies show that combined CCR7 and CXCR4 expression is also correlated with lymph nodes metastasis in breast cancer [38, 140, 141], gastric cancer [142], cervical cancer [143], and melanoma [38]. High levels of CCR7 are consistently expressed in breast tumor cells along with CXCR4, though CCR7 is also expressed in normal mammary epithelial cells [38]. Similar to CXCR4, CCR7-CCL21 interactions induces directional invasion of breast tumor cells, pseudopodia formation, and actin polymerization which increases the invasiveness of tumor cells [38]. Since both CCL21 and CXCL12 are highly expressed in the lymph nodes and receptor-ligand interactions of both chemokines promote invasiveness, it is likely that the two ligands work together to promote metastasis to the lymph nodes [38, 140].

CCR7 appears to play an especially important role in leukemias and lymphomas. Due to their lymphoid origin, many leukemias and lymphomas highly express CCR7 and experience frequent metastasis to the lymph nodes [144]. B cell malignancies with widespread dissemination to the lymph nodes, such as B-cell chronic lymphocytic leukemia (B-CLL) [145], express high levels of CCR7, which mediates B cell entry into the lymphoid tissue, while neoplasms that experience little metastasis to the lymph nodes, such as multiple myeloma or hairy cell leukemia [145], express low levels of CCR7 and low to moderate levels of CXCR4 [144].

In addition, studies suggest that CCR7 mediates metastasis of T-cell leukemia to the central nervous system (CNS). Buonomici *et al*. show that Notch1 signaling, active and mutated in most T-ALL patients [146], upregulates CCR7 expression in T-ALL. Expressing oncogenic Notch 1 causes mice to develop characteristic pathological features of T-ALL and infiltration of the leptomeningeal spaces of the brain, demonstrating that oncogenic Notch1 is capable of inducing T-ALL and targeting transformed cells to the CNS [147]. Analysis of primary T-ALL samples as well as T-ALL cell-lines containing Notch1-activating mutations have CCR7 upregulation caused by Notch1 signaling [147]. CCR7 also plays an important role in squamous cell carcinoma of the head and neck (SCCHN). Studies show CCR7 upregulation in SCCHN is correlated with lower survival due to increased metastasis [148]. CCR7 mediates SCCHN metastasis by activating integrin αvβ3 [139], which facilitates adhesion of cancer cells to or migration through the extracellular matrix. In additions, CCR7 mediates cell survival of metastatic SCCHN cell lines by phosphorylation of Akt in a PI3K-dependent fashion [149-151]. CCR7-CCL19 activation of the PI3K/Akt/mTOR signal pathway subsequently activates the downstream signal molecule NF-κB [149]. CCR7-CCL19 interactions induce phosphorylation of IκBα, causing NF-κB to translocate to the nucleus and raise the DNA-binding capacity of NF-κB [149]. Inhibition of CCR7, PI3K, Akt and mTOR successfully stops phosphorylation and DNA-binding. Moreover, CCR7 and NF-κB expression in patient samples directly correlates and is also associated with increased lymph node metastasis and clinical progression [149]. Thus, CCR7 enhances survival of metastatic SCCHN by activating NF-κB via the PI3K/Akt/mTOR signal pathway [149].

#### c CCR9-CCL25

In normal physiological conditions, CCR9 functions in mucosal immunity and lymphocyte trafficking during T-cell development [152]. Its ligand CCL25, or TECK, is expressed in the small intestine and thymus [153]. As migration of CCR9-postive lymphocytes to the small intestine relies on chemoattractive effects of CCL25 and the action of αβ integrin heterodimers, studies suggest CCR9-CCL25 also mediates melanoma metastasis to the small intestine. Most tumors of the small intestine, which are rare and account for just 2% of all gastrointestinal tumors, are metastasis from melanoma [154-156]. Studies investigating melanoma metastasis demonstrate that CCR9 is highly expressed in melanoma cells and small intestinal metastasis [157] and that functional expression of CCR9 on melanoma cells mediates migration to the small intestine, explaining their preferential migration to the small intestine [158].

Some breast cancer cell lines also express CCR9 [159]. A recent study suggests that CCR9 may promote metastasis in breast cancer by enhancing cell migration, increasing matrix-metalloproteinase-9 (MMP-9) expression via CCL25 and supporting tumor cell survival in metastatic sites [159]. Certain ovarian cancer cell lines and tumors also express CCR9 [160]; however, further research is needed to elucidate the role of CCR9 in other cancers and to determine the mechanism by which it promotes metastasis.

#### d CCR10-CCL27/CCL28

The chemokine receptor CCR10 binds ligand CCL27, which is highly expressed in both normal and inflammatory skin [161]. In the skin, CCL27 recruits leukocyte antigen CLA+ T cells which express chemokine receptor CCR10 [83]. Studies show that malignant melanoma expresses high levels of CCR10 in addition to CXCR4 and CCR7 [162]. Melanoma shares a similar metastatic phenotype to breast cancer, which also expresses high levels of CXCR4 and CCR7, but unlike breast cancer, experiences frequent metastasis to the skin [38]. Given the role of CCR10-CCL27 in leukocyte migration to the skin, it is likely that CCR10 expression in melanoma induces metastasis to the skin. However, another study suggests that CCR10 expression in melanoma promotes metastasis to the lymph nodes in addition to enhancing invasion, growth, and immune escape of tumor cells [163]. CCR10 and its ligand CCL27 are also upregulated in cutaneous squamous cell carcinoma, and its expression is correlated with progression of the cancer [164].

#### e CXCR3-CXCL9, CXCL10, CXCL11

CXCR3 expression in cutaneous malignant melanoma correlates with tumor progression [165], and is constitutively expressed in several human melanoma cell lines and mouse B16F10 melanoma cell line [166]. Loss of CXCR3 expression in mouse B16F10 melanoma decreases metastasis to the lymph nodes by ~15%, suggesting that CXCR3 may play a role in lymph node metastasis [166]. CXCR3 is also constitutively expressed in lung adenocarcinoma; however, CXCR3 expression is not significantly correlated with lymph node metastasis [167]. Some colon cancers also constitutively express CXCR3, and patients that express CXCR3 in tumors experience worse prognosis than those without CXCR3 [168]. Kawada *et. al* demonstrate that expressing CXCR3 in a colon cancer cell line increases *in vivo* metastasis to the lymph nodes, but not to the liver or lungs [168]. Cambien *et. al*, on the other hand, report that *in vivo* activation of CXCR3 increases metastasis to the lungs [169]. Additional studies suggest that CXCR3 activation in colon cancer synergistically enhances CXCR4-mediated tumor cell migration to the lymph nodes, liver, and lungs [170]. Thus, the function of CXCR3 in colon cancer may depend on its cooperation with other expressed chemokine receptors such as CXCR4 and CCR7.

In osteosarcoma, studies suggest that CXCR3 and its ligands induce metastasis to the lungs and later stimulate growth and expansion of the metastases [171]. Treating mice with CXCR3 antagonist AMG487 significantly decreases lung metastases and reduces tumor expansion within the lungs in two murine models of osteosarcoma [171]. CXCR3 inhibition similarly reduces lung metastasis in a metastatic breast cancer murine model [172]. These studies suggest that CXCR3 may play an important role in mediating metastasis to the lungs.

#### f CXCR5-CXCL13

CXCR5, along with CXCR4 and CCR7, also helps regulate B cell entry into secondary lymphoid tissues and their homing to T cell and B cell zones within lymphoid tissues [173, 174]. Studies show certain lymphomas express CXCR5 along with CCR7 in malignancies which experience frequent metastasis to the lymph nodes suggesting a role of CXCR5 in lymph node metastasis [144]. The expression of CXCR5 is especially high in chronic lymphocytic leukemia and mantle lymphoma [144]. Consistent CXCR5 upregulation is also observed in squamous cell carcinomas, suggesting that CXCR5/CXCL13 interactions may serve as a novel complementary pathway mediating metastasis to the lymph nodes [131]. CXCR5 may also mediate metastasis of neuroblastoma cells to the bone marrow through interactions with CCL16 expressing stromal cells [175]. However, further research is required to elucidate the role of CXCR5 in other malignancies and determine the mechanisms by which it promotes metastasis.

### Chemokine Receptors and Brain metastasis

Brain metastasis is one of the major causes of cancer related deaths in patients. To develop novel therapeutic approaches for patients with brain metastasis, it is important to understand the biology of the metastatic lesions in the brain and the role of the brain microenvironment in promoting brain metastasis. Since chemokine receptors have already been shown to play a role in metastasis, there is little doubt about their role in brain metastasis as well. The chemokine receptor CXCR4 is overexpressed in brain metastatic sections of patients with non-small cell lung cancer [176, 177]. Also, CXCR4 is overexpressed in brain metastatic sections of patients with colorectal cancer [165]. Furthermore, another study demonstrates the importance of CXCR4 in promoting invasion through human brain microvascular endothelial cells in breast cancer *in vitro* [178]. The chemokine receptor CX3CR1 has been also associated with brain metastasis in axillary node positive primary breast cancer [179]. Finally, CCR4 has been shown to play a role in melanoma metastasis to the brain [180].

The chemokine receptor CCR7 interacts with CCL19 and mediates T-cell acute lymphoblastic leukemia (ALL) metastasis to the central nervous system. A lymphoid Rag2^−/−^IL2rg^−/−^ mouse transplanted with CCR7-positive (CEM/ CCR7^+^) human T-ALL cell line results in CNS infiltration and reduced survival in comparison to mice transplanted with a CCR7-deficient (DND41/CCR7^−^) T-ALL cell line. Meanwhile, expression of CCR7 in DND41 cells is sufficient to induce brain and spinal cord infiltration, while silencing CCR7 successfully inhibits T-ALL CNS metastasis. However, CCR7 appears to regulate CNS infiltration only in Notch-1-induced T-cell malignancy, as CCR7 inhibition fails to suppress CNS infiltration in B-cell models. Overall, these data demonstrate that in addition to promoting metastasis to the lymph nodes, CCR7 also regulates metastasis of T-cell leukemia to the brain and central nervous system [147]. These studies suggest the important role of the chemokine receptors in brain metastasis.

## CONCLUSION

A number of studies posit the role of chemokine and chemokine receptors in various stages of tumor progression. Chemokines and their receptors facilitate tumor growth by promoting EMT as well as angiogenesis. The expression of certain chemokine receptors on the surface of the cancer cells promotes metastasis and organ specific metastasis. CXCR4 is one of the well-studied chemokine receptors. In 2010, the U.S Food and Drug Administration approved the CXCR4 inhibitor plerixafor in patients with non-Hodgkin lymphoma (NHL) and multiple myeloma (MM). Moreover, while some chemokine receptors, such as CXCR4 and CCR7, have been extensively studied, additional studies are needed to examine the role of other chemokines in tumor progression and metastasis. Future studies should focus on elucidating the molecular mechanism and signaling pathways through which chemokines mediate tumor progression and metastasis. Furthermore, it will be interesting to explore the prospect of a combination therapy of chemokine receptor antagonists with other chemotherapeutic agents to determine the effect on survival in patients. Ultimately, chemokines and chemokine receptors may serve as useful therapeutic targets in preventing tumor progression and metastasis and thus help in improving survival in cancer patients.
